# Effects of coalescence and isospin symmetry on the freezeout of light nuclei and their anti-particles

**DOI:** 10.1038/s41598-021-99455-x

**Published:** 2021-10-12

**Authors:** M. Waqas, G. X. Peng, Fu-Hu Liu, Z. Wazir

**Affiliations:** 1 School of Nuclear Science and Technology, University of Chinese Academy of Sciences, Beijing, 100049 China; 2 Theoretical Physics Center for Science Facilities, Institute of High Energy Physics, Beijing, 100049 China; 3 Synergetic Innovation Center for Quantum Effects & Application, Hunan Normal University, Changsha, 410081 China; 4 Institute of Theoretical Physics & State Key Laboratory of Quantum Optics and Quantum Optics Devices, Shanxi University, Taiyuan, 030006 China; 5 Collaborative Innovation Center of Extreme Optics, Shanxi University, Taiyuan, 030006 China; 6 Department of Physics, Ghazi University, Dera Ghazi Khan, Pakistan

**Keywords:** Theoretical nuclear physics, Phenomenology

## Abstract

The transverse momentum spectra of light nuclei (deuteron, triton and helion) produced in various centrality intervals in Gold–Gold (Au–Au), Lead–Lead (Pb–Pb) and proton–Lead (p–Pb) collisions, as well as in inelastic (INEL) proton–proton (p–p) collisions are analyzed by the blast wave model with Boltzmann Gibbs statistics. The model results are nearly in agreement with the experimental data measured by STAR and ALICE Collaborations in special transverse momentum ranges. We extracted the bulk properties in terms of kinetic freezeout temperature, transverse flow velocity and freezeout volume. It is observed that deuteron and anti-deuteron freezeout later than triton and helion as well as their anti-particles due to its smaller mass, while helion and triton, and anti-helion and anti-triton freezeout at the same time due to isospin symmetry at higher energies. It is also observed that light nuclei freezeout earlier than their anti-nuclei due to the large coalescence of nucleons for light nuclei compared to their anti-nuclei. The kinetic freezeout temperature, transverse flow velocity and kinetic freezeout volume decrease from central to peripheral collisions. Furthermore, the transverse flow velocity depends on mass of the particle which decreases with increasing the mass of the particle.

## Introduction

A new form of matter, the so called quark-gluon plasma (QGP) is produced at high temperatures and energy densities in relativistic heavy ion collisions. In nuclear physics, among the various probes, charmonia (i.e. $j/\psi$ suppression) are very sensitive probes of the characteristics of QGP^1,2^. Charmonia are the bound states of charm–anticharm $(c{\bar{c}})$ quarks, and they are formed early in the heavy ion collisions and their yields are expected to be suppressed in the medium. Numerous theoretical and experimental studies^3–5^ enrich our understanding of quarkonia as probes of QGP. The $j/\psi$ suppression was measured at SPS^6,7^ and was termed as ‘anomalous’ $j/\psi$ suppression and it was considered a hint for QGP. This matter is formed in the early stage of collisions that survives for a very short period of time ($\sim$ 7–10 fm/c), after which the QGP gets transformed rapidly to a system of hadron gas. Due to multi-partonic interactions throughout the evolution time in the collisions, the information about the initial condition of the system get mostly lost. The final state behavior of such colliding system can be attained from the measurement of the number as well as the identity of the produced particles along with their energy and momentum spectra. The final state information are very useful to understand the particle production mechanisms and the nature of the matter created in these high energy collisions.

Temperature is one of the most crucial factor in sub-atomic physics. There are different types of temperatures present in literature^7–12^. Chemical freezeout temperature ($T_{ch}$), which describes the excitation degree of interacting system at the stage of chemical freezeout. At chemical freezeout the chemical components (relative fraction) of the particles are invariant. The excitation degree of the interacting system at the stage of thermal or kinetic freezeout is described by the kinetic freezeout temperature ($T_0$). At kinetic freezeout, the transverse momentum spectra of the particles are no longer changed. Another type of temperature is the effective ($T_{eff}$). It is not a real temperature but is related to the particle mass and can be extracted from the transverse momentum spectra by using various distribution laws such as standard (Boltzmann, Bose-Einstein and Fermi-Dirac), Tsallis, and so forth.

The chemical freezeout temperature is usually obtained from the particle ratio^13–15^. Due to chemical equilibrium being meanwhile or earlier than the kinetic equilibrium, the chemical freezeout temperature is equal or higher than the kinetic freezeout temperature. The effective temperature, due to mass and flow velocity are also larger than the kinetic freezeout temperature^16,17^. Due to more violent interactions in central collisions, both the chemical freezeout temperature and effective temperature are larger in central collisions than in the peripheral collisions. However, the situation for kinetic freezeout temperature is not clear. several literatures claim larger $T_0$ in central collisions^18–23^ which decrease towards periphery, while several claims larger $T_0$ in peripheral collisions^24–27^ which decrease towards the central collisions. In addition, volume is also very important parameter in high energy collisions. The volume occupied by the ejectiles when the correlative interactions become negligable and the only force they experience is Coulombic force, is said to be kinetic freezeout volume (*V*). Most of the literatures agreed to the larger *V* as well as the transverse flow velocity ($\beta _T$) in central collisions which decrease towards periphery.

Freezeout scenario is very important in high energy collisions. Different freezeout scenarios are discussed in literature at different stages of the freezeout, but we will focus on kinetic freezeout scenarios in the present work. There are several kinetic freezeout scenarios in literature^22,23,28–32^ which include single, double, triple and multiple freezeout scenario. In the study of production of light nuclei, it is expected that the freezeout of the particles may also be dependent on the nucleon coalescence and isospin symmetry at higher energies. However it is hard to say that the coalescence and isospin symmetry play a role in $j/\psi$ suppression because its production mechanism is very complicated.

The transverse momentum spectra ($p_T$) of the particles are very important observables because they give very crucial information about the equilibrium dynamics and the anisotropy of the produced system in heavy ion collisions^31^. In the present work, we will analyze the $p_T$ spectra of deuteron (*d*), anti-deuteron ($\bar{d}$), triton (*t*), anti-triton (${\bar{t}}$), helion ($^3{\text{He}}$) and anti-helion ($^3{\bar{\text{He}}}$) in Gold–Gold (Au–Au), Lead–Lead (Pb–Pb), Proton–Lead (p–Pb) and proton–proton (p–p) collisions.

## The method and formalism

A few methods can be used for the extraction of $T_0$ and $\beta _T$, including but not limited to, (1) the blast-wave model with Boltzmann–Gibbs statistics (BGBW)^33–35^, (2) the blast-wave model with Tsallis statistics^36^, (3) an alternative method using the Boltzmann distribution^29,35–42^, and (4) the alternative method using the Tsallis distribution^43,44^. It is noteworthy that $T_0$ is the intercept in the linear relation *T*-$m_0$ in alternative method, where $m_0$ is the rest mass; and $\beta _T$ is the slope in the linear relation $<p_T>$-${\bar{m}}$, where $<p_T>$ is the mean transverse momentum and ${\bar{m}}$ is the mean moving mass (i.e., the mean energy).

Reference^45^ confirms that the above methods are harmonious. Among these methods, the first method is the most direct with fewer parameters, though it has been revised in various ways and applied to other quantities^46–50^. We have used the first method, i.e., BGBW in the present work. Due to their coherence, other models will not be used^45^.

BGBW is a phenomenological model which is used for the spectra of hadrons based on flowing local thermal sources with global variables of temperature, volume and transverse flow velocity. According to^33–35^, the $p_T$ distribution of BGBW can be written as

1 $$\begin{aligned} f(p_T)=&\frac{1}{N}\frac{dN}{dp_T} = \frac{1}{N}\frac{gV}{(2\pi )^2} p_T m_T \int _0^R rdr \nonumber \\&\times I_0 \bigg [\frac{p_T \sinh (\rho )}{T} \bigg ] K_1 \bigg [\frac{m_T \cosh (\rho )}{T} \bigg ], \end{aligned}$$

where *N* is the number of particles, *g* represents the degeneracy factor of the particle (which is different for different particles, based on $g_n$=2$S_n$+1, while $S_n$ is the spin of the particle), *V* is the freezeout volume, $m_T$ is the transverse mass ($m_T=\sqrt{p_T^2+m_0^2}$), $I_0$ and $K_1$ are the modified Bessel functions, $\rho = \tanh ^{-1} [\beta (r)]$, $\beta (r)= \beta _S(r/R)^{n_0}$ is the transverse radial flow of the thermal source at radius $$0 \le \ r \le \ $$ $R$ with surface velocity $\beta _S$ and $n_0$=1^36^. In general, $\beta _T=(2/R^2)\int _0^R r\beta (r)dr=2\beta _S/(n_0+2)=2\beta _S/3$.

Equation (1) can be used for the fitting of $p_T$ spectra to obtain the parameters $T_0$, *V* and $\beta _T$. It should be noted that Eq. (1) can be only valid in a narrow $p_T$ range i.e: they describe only soft excitation process. However we have to consider the hard scattering process for the spectra in a wide $p_T$ range. In general, the contribution of hard process can be parameterized to an inverse power law [51–453], i.e., Hagedorn function^54,55^

2 $$\begin{aligned} f_0(p_T)=\frac{1}{N}\frac{dN}{dp_T}= Ap_T \bigg ( 1+\frac{p_T}{ p_0} \bigg )^{-n}, \end{aligned}$$

which is resulted from the calculus of quantum chromodynamics (QCD)^51–53^, where $p_0$ and *n* are the free parameters and *A* is the normalization constant that depends on $p_0$ and *n*.

In order to describe a wide $p_T$ range, the superposition of both the soft and hard process can be used, which is

3 $$\begin{aligned} f_0(p_T)=kf_S(p_T) +(1-k)f_H(p_T), \end{aligned}$$

where *k* shows the contribution fraction of the first component (soft excitation), while $(1-k)$ represents the contribution fraction of the second component (hard scattering) in Eq. (3), and according to Hagedorn model^55^ the usual step function can be also be used for the superposition of soft and hard components.

According to Hagedorn thermal model^55^, the two-component BGBW distribution function can also be structured by using the usual step function,

4 $$\begin{aligned} f_0(p_T)&=\frac{1}{N}\frac{dN}{dp_T}=A_1\theta (p_1-p_T)f(p_T) \nonumber \\&\quad + A_2 \theta (p_T-p_1) f(p_T), \end{aligned}$$

where $A_1$ and $A_2$ are the constants which give the two components to be equal to each other at $p_T$ = $p_1$.

## Results and discussion

**Figure 1:**
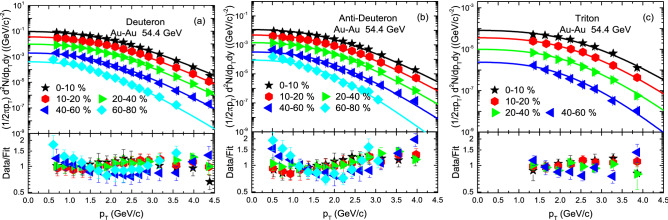
The transverse momentum ($p_T$) spectra of (**a**) deutron, (**b**) anti-deutron and (**c**) triteron produced in $|y|<0.5$ in different centrality classes in Au–Au collisions at 54.4 GeV. The symbols are the experimental data of the STAR Collaboration^56^ and the curves are our fitting by using BGBW statistics. Each panel is followed by the ratio of the data/fit.

**Figure 2:**
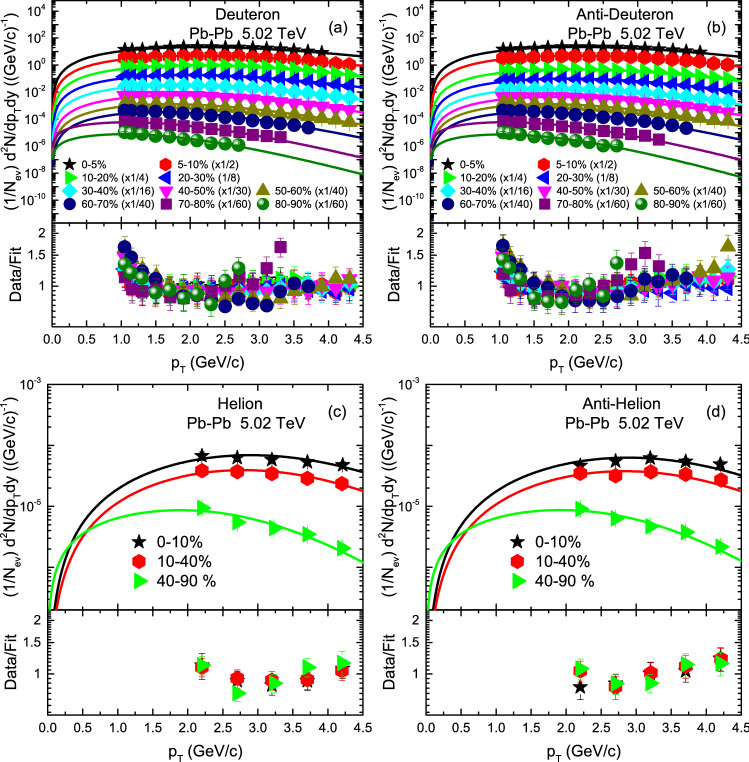
Transverse momentum spectra of *d*, ${\bar{d}}$, $^3{\text{He}}$ and $^3{\bar{\text{He}}}$ in $|y|<0.5$ produced in different centrality intervals in Pb–Pb collisions at 5.02 TeV. The symbols represent the experimental data measured by the ALICE Collaboration^57^, while the curves are our fitted results by using BGBW statistics, Eq. (1). Each panel is followed by the ratio of the data/fit.

The transverse momentum ($p_T$) spectra, $(1/2\pi p_T)d^2N/dp_Tdy$, of *d*, ${\bar{d}}$ and *t* produced in AuAu collisions at 54.4 GeV are analyzed by BGBW statistics in different centrality classes are demonstrated in Fig. 1. The symbols represent the experimental data of the STAR Collaboration measured in the mid-rapidity range $|y|<0.5$ and the solid curve are the results of our fitting by using Eq. (1). The well approximate description of the model result to the experimental data of the STAR Collaboration^56^ in the special $p_T$ range can be seen. The event centralities and the values of free parameters are listed in Table 1. The free parameters include kinetic freezeout temperature ($T_0$), transverse flow velocity ($\beta _T$), kinetic freezeout volume (*V*), normalization constant ($N_0$), $\chi ^2$ and the degree of freedom (dof). Each panel is followed by the results of its corresponding ratio of the data/fit. Figure 2 demonstrates the $p_T$ spectra, $(1/N_{ev}) d^2N/dp_Tdy$ of *d*, ${\bar{d}}$, $^3{\text{He}}$ and $\bar{^{3}{\text{He}}}$ in various centrality classes in p–Pb collisions at 5.02 TeV. The symbols stands for the experimental data of the ALICE Collaboration^57^ by the Large Hadron Collider (LHC) and the solid curve represent our fitting results by using the BGBW statistics. In Fig. 2 some spectra are scaled; such as the spectra of *d* and $\bar{d}$ in centrality intervals 5–10%, 10–20%, 20–30%, 30–40%, 40–50%, 50–60%, 60–70%, 70–80% and 90–90% are multiplied by 1/2, 1/4, 1/8, 1/16, 1/30, 1/40, 1/40, 1/60 and 1/60, respectively.

**Table 1:** List of the parameters.

Collisions	Centrality	Particle	$T_0$ (GeV)	$\beta _T (c)$	$V (fm^3)$	$N_0$	$\chi ^2$/ dof
Figure 1 Au–Au 54.4 GeV	0–10%	*d*	$0.118\pm 0.006$	$0.460\pm 0.009$	$3500\pm 183$	$4.5\times 10^{-4}\pm 5\times 10^{-5}$	10/11
	10–20%	–	$0.113\pm 0.006$	$0.452\pm 0.008$	$3350\pm 176$	$1.7\times 10^{-4}\pm 5\times 10^{-5}$	4/11
	20–40%	–	$0.109\pm 0.005$	$0.440\pm 0.007$	$3200\pm 150$	$4\times 10^{-5}\pm 5\times 10^{-6}$	11/11
	40–60%	–	$0.105\pm 0.005$	$0.427\pm 0.009$	$3000\pm 207$	$9\times 10^{-6}\pm 5\times 10^{-7}$	14/11
	60–80%	–	$0.101\pm 0.006$	$0.388\pm 0.010$	$2800\pm 210$	$1.6\times 10^{-6}\pm 6\times 10^{-7}$	17/9
Au–Au 54.4 GeV	0–10%	$\bar{d}$	$0.110\pm 0.005$	$0.460\pm 0.011$	$3200\pm 170$	$5.9\times 10^{-5}\pm 4\times 10^{-6}$	12/10
	10–20%	–	$0.105\pm 0.006$	$0.453\pm 0.010$	$3100\pm 180$	$2.45\times 10^{-5}\pm 5\times 10^{-6}$	10/10
	20–40%	–	$0.100\pm 0.005$	$0.440\pm 0.008$	$2900\pm 180$	$7.2\times 10^{-6}\pm 6\times 10^{-7}$	22/10
	40–60%	–	$0.097\pm 0.006$	$0.427\pm 0.011$	$2760\pm 170$	$1.6\times 10^{-6}\pm 4\times 10^{-7}$	44/10
	60–80%	–	$0.092\pm 0.006$	$0.388\pm 0.012$	$2650\pm 140$	$3.7\times 10^{-7}\pm 5\times 10^{-8}$	34/8
Au–Au 54.4 GeV	0–10%	*t*	$0.130\pm 0.005$	$0.420\pm 0.010$	$2700\pm 190$	$1.3\times 10^{-6}\pm 6\times 10^{-7}$	16/3
	10–20%	–	$0.126\pm 0.005$	$0.400\pm 0.012$	$2500\pm 150$	$5.5\times 10^{-7}\pm 4\times 10^{-8}$	2/3
	20–40%	–	$0.122\pm 0.006$	$0.388\pm 0.009$	$2360\pm 128$	$1.5\times 10^{-7}\pm 3\times 10^{-8}$	8/3
	40–60%	–	$0.118\pm 0.005$	$0.363\pm 0.012$	$2200\pm 146$	$3.3\times 10^{-8}\pm 5\times 10^{-9}$	16/3
Figure 2 Pb–Pb 5.02 TeV	0–5%	*d*	$0.156\pm 0.005$	$0.545\pm 0.009$	$5100\pm 172$	$0.077\pm 0.004$	1/14
	5–10%	–	$0.153\pm 0.004$	$0.540\pm 0.012$	$4940\pm 160$	$0.037\pm 0.005$	3/14
	10–20%	–	$0.150\pm 0.005$	$0.535\pm 0.012$	$4710\pm 160$	$0.015\pm 0.004$	4/14
	20–30%	–	$0.147\pm 0.005$	$0.527\pm 0.009$	$4500\pm 167$	$0.006\pm 0.0004$	5/14
	30–40%	–	$0.145\pm 0.005$	$0.510\pm 0.009$	$4300\pm 167$	$0.0023\pm 0.0004$	6/14
	40–50%	–	$0.143\pm 0.005$	$0.500\pm 0.009$	$4200\pm 167$	$7.5\times 10^{-4}\pm 3\times 10^{-5}$	6/14
	50–60%	–	$0.140\pm 0.005$	$0.480\pm 0.012$	$4100\pm 200$	$2.3\times 10^{-4}\pm 3\times 10^{-5}$	21/14
	60–70%	–	$0.137\pm 0.006$	$0.450\pm 0.010$	$3910\pm 190$	$6.3\times 10^{-5}\pm 4\times 10^{-6}$	29/11
	70–80%	–	$0.133\pm 0.005$	$0.440\pm 0.012$	$3500\pm 160$	$2.3\times 10^{-5}\pm 3\times 10^{-6}$	16/9
	80–90%	–	$0.130\pm 0.006$	$0.420\pm 0.010$	$3300\pm 150$	$3\times 10^{-6}\pm 5\times 10^{-7}$	12/6
Pb–Pb 5.02 TeV	0–5%	$\bar{d}$	$0.148\pm 0.005$	$0.545\pm 0.009$	$4700\pm 181$	$0.086\pm 0.005$	3/14
	5–10%	–	$0.145\pm 0.006$	$0.540\pm 0.012$	$4500\pm 190$	$0.04\pm 0.006$	5/14
	10–20%	–	$0.142\pm 0.005$	$0.535\pm 0.011$	$4300\pm 172$	$0.0166\pm 0.004$	5/14
	20–30%	–	$0.140\pm 0.005$	$0.527\pm 0.012$	$4100\pm 150$	$0.007\pm 0.0004$	6/14
	30–40%	–	$0.137\pm 0.006$	$0.510\pm 0.010$	$3900\pm 170$	$0.0026\pm 0.0004$	9/14
	40–50%	–	$0.133\pm 0.005$	$0.500\pm 0.012$	$3700\pm 180$	$8.5\times 10^{-4}\pm 5\times 10^{-5}$	12/14
	50–60%	–	$0.130\pm 0.004$	$0.480\pm 0.010$	$3500\pm 156$	$2.6\times 10^{-4}\pm 4\times 10^{-5}$	26/14
	60–70%	–	$0.128\pm 0.005$	$0.450\pm 0.008$	$3300\pm 160$	$7.2\times 10^{-5}\pm 6\times 10^{-6}$	22/11
	70–80%	–	$0.125\pm 0.004$	$0.440\pm 0.010$	$3100\pm 180$	$2.6\times 10^{-5}\pm 5\times 10^{-6}$	16/9
	80–90%	–	$0.122\pm 0.005$	$0.420\pm 0.012$	$2900\pm 140$	$3.2\times 10^{-6}\pm 4\times 10^{-7}$	15/6
Pb–Pb 5.02 TeV	0–10%	$^3\text{He}$	$0.164\pm 0.005$	$0.510\pm 0.007$	$4400\pm 160$	$1.5\times 10^{-7}\pm 4\times 10^{-8}$	3/1
	10–40%	–	$0.160\pm 0.005$	$0.500\pm 0.008$	$4200\pm 140$	$9\times 10^{-8}\pm 4\times 10^{-9}$	2/1
	40–100%	–	$0.156\pm 0.004$	$0.470\pm 0.010$	$4000\pm 180$	$4.7\times 10^{-8}\pm 4\times 10^{-9}$	10/1
Pb–Pb 5.02 TeV	0–10%	$\bar{^{3}{\text{He}}}$	$0.158\pm 0.004$	$0.510\pm 0.007$	$4000\pm 140$	$1.4\times 10^{-7}\pm 4\times 10^{-8}$	4/1
	10–40%	–	$0.154\pm 0.005$	$0.505\pm 0.010$	$3800\pm 166$	$9.5\times 10^{-8}\pm 4\times 10^{-8}$	3/1
	30–100%	–	$0.151\pm 0.005$	$0.475\pm 0.009$	$3600\pm 155$	$5.3\times 10^{-8}\pm 4\times 10^{-9}$	4/1
Figure 3 p–Pb 5.02 TeV	0–10%	*d*	$0.148\pm 0.006$	$0.480\pm 0.008$	$4720\pm 170$	$1.25\times 10^{-4}\pm 4\times 10^{-5}$	12/7
	10–20%	–	$0.143\pm 0.006$	$0.470\pm 0.011$	$4550\pm 160$	$4.5\times 10^{-5}\pm 5\times 10^{-6}$	18/7
	20–40%	–	$0.139\pm 0.006$	$0.440\pm 0.007$	$4400\pm 186$	$1.7\times 10^{-5}\pm 6\times 10^{-6}$	13/4
	40–60%	–	$0.135\pm 0.005$	$0.430\pm 0.008$	$4200\pm 179$	$5\times 10^{-6}\pm 5\times 10^{-7}$	21/4
	60–100%	–	$0.131\pm 0.005$	$0.400\pm 0.010$	4130$\pm 180$	$8\times 10^{-7}\pm 6\times 10^{-8}$	35/4
p–Pb 5.02 TeV	0–10%	$\bar{d}$	$0.140\pm 0.005$	$0.480\pm 0.012$	$4400\pm 184$	$1.31\times 10^{-4}\pm 6\times 10^{-5}$	8/7
	10–20%	–	$0.136\pm 0.006$	$0.470\pm 0.010$	$4200\pm 140$	$4.4\times 10^{-5}\pm 6\times 10^{-6}$	18/5
	20–40%	–	$0.132\pm 0.005$	$0.440\pm 0.009$	$4000\pm 160$	$1.75\times 10^{-5}\pm 7\times 10^{-6}$	8/5
	40–60%	–	$0.128\pm 0.004$	$0.430\pm 0.008$	$3800\pm 154$	$5.5\times 10^{-6}\pm 7\times 10^{-7}$	21/4
	60–100%	–	$0.122\pm 0.004$	$0.400\pm 0.010$	3600$\pm 168$	$1\times 10^{-6}\pm 4\times 10^{-7}$	16/4
p–Pb 5.02 TeV	0–10%	$(^3\text{He}+\bar{^3\text{He}})/2$	$0.154\pm 0.006$	$0.470\pm 0.008$	$4000\pm 191$	$5\times 10^{-8}\pm 4\times 10^{-9}$	4/–
	10–20%	–	$0.150\pm 0.005$	$0.443\pm 0.011$	$3800\pm 166$	$1.4\times 10^{-8}\pm 5\times 10^{-9}$	3/–
	20–40%	–	$0.146\pm 0.005$	$0.413\pm 0.009$	$3600\pm 165$	$5\times 10^{-9}\pm 3\times 10^{-10}$	26/–
	40–100%	–	$0.142\pm 0.005$	$0.410\pm 0.011$	3400$\pm 150$	$7\times 10^{-10}\pm 3\times 10^{-11}$	1/–
Figure 4 p–p 7 TeV	–	*d*	$0.090\pm 0.006$	$0.420\pm 0.008$	$3000\pm 158$	$2\times 10^{-6}\pm 4\times 10^{-7}$	141/17
	–	$\bar{d}$	$0.080\pm 0.005$	$0.420\pm 0.007$	$2600\pm 145$	$2\times 10^{-6}\pm 4\times 10^{-7}$	81/16
	–	*t*	$0.105\pm 0.004$	$0.350\pm 0.009$	$2000\pm 155$	$1.3\times 10^{-10}\pm 3\times 10^{-11}$	0.02/–
	–	$\bar{t}$	$0.095\pm 0.004$	$0.350\pm 0.008$	$2000\pm 145$	$1.6\times 10^{-10}\pm 4\times 10^{-11}$	0.02/–
	–	$^3\text{He}$	$0.105\pm 0.004$	$0.300\pm 0.007$	$2000\pm 155$	$1.3\times 10^{-10}\pm 3\times 10^{-11}$	0.6/–
	–	$^3{\bar{\text{He}}}$	$0.095\pm 0.005$	$0.350\pm 0.007$	$2000\pm 145$	$6\times 10^{11}\pm 4\times 10^{-12}$	38/–

– is used in some places instead of dof. In fact it is not the fit result. if dof < 0, the we put – instead of negative values.

**Figure 3:**
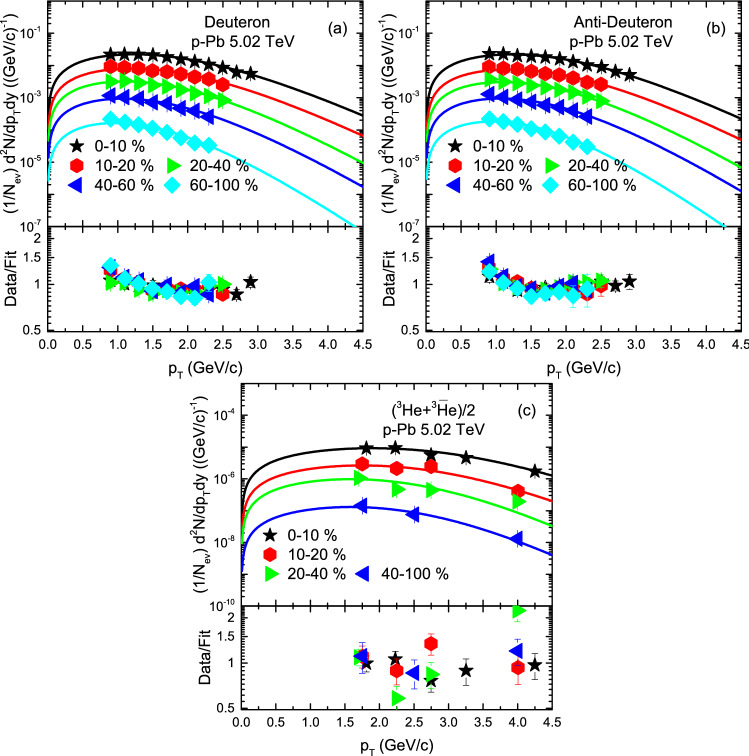
Transverse momentum spectra of (**a**) *d*, (**b**) ${\bar{d}}$ and (**c**) ($^3{\text{He}}$+$\bar{^{3}{\text{He}}}$)/2 produced in various centrality bins in p–Pb collisions at 5.02 TeV. The symbols represent the experimental data measured by ALICE Collaboration^58,59^, while the curves are our fitted results by using BGBW statistics, Eq. (1). Each panel is followed by the ratio of the data/fit.

Figure 3 is similar to Fig. 2, but it shows the $p_T$ spectra of *d*, ${\bar{d}}$ and ($^3{\text{He}}$+$\bar{^{3}{\text{He}}}$)/2 produced in different centrality intervals in p–Pb collisions at 5.02 TeV. The symbols show the experimental data measured by the ALICE Collaboration in the rapidity region $$-1 \le \ y \le \ $$1 and − 1 $y$ 0 respectively, and the curves are the results of our fitting by using Eq. (1). The well approximate description of the model results to the experimental data of the ALICE Collaboration^58,59^ in the special $p_T$ range can be seen.

**Figure 4:**
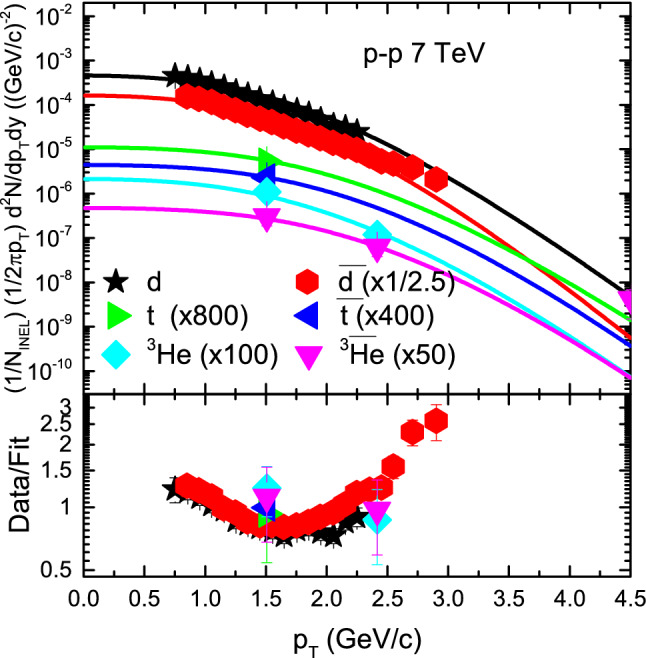
Figure 4 is similar to Figs. 2 and 3, but it shows $p_T$ spectra of *d*, ${\bar{d}}$, *t*, ${\bar{t}}$, $^3{\text{He}}$ and $^3{\bar{\text{He}}}$ in $|y|<0.5$ produced in INEL p–p collisions at 7 TeV. The symbols represent the experimental data measured by the ALICE Collaboration^60^, while the curves are our fitted results by using BGBW statistics, Eq. (1). Each panel is followed by the ratio of the data/fit.

In Fig. 4 the $p_T$ spectra of *d*, ${\bar{d}}$, *t*, ${\bar{t}}$, $^3{\text{He}}$ and $\bar{^{3}{\text{He}}}$ in inelastic (INEL) p–p collisions at 7 TeV are presented. The symbols represent the experimental data of the ALICE Collaboration by the LHC in the rapidity interval of $|y|<0.5$ and the results of our fitting is represented by curve. The spectra of ${\bar{d}}$, *t*, ${\bar{t}}$, $^3 \text{He}$ and $\bar{^{3}{\text{He}}}$ are multiplied by 1/2.5, 800, 400, 100 and 50, respectively. One can see the well approximate description of the model results to the experimental data of the ALICE Collaboration^60^ in the special $p_T$ range.

**Figure 5:**
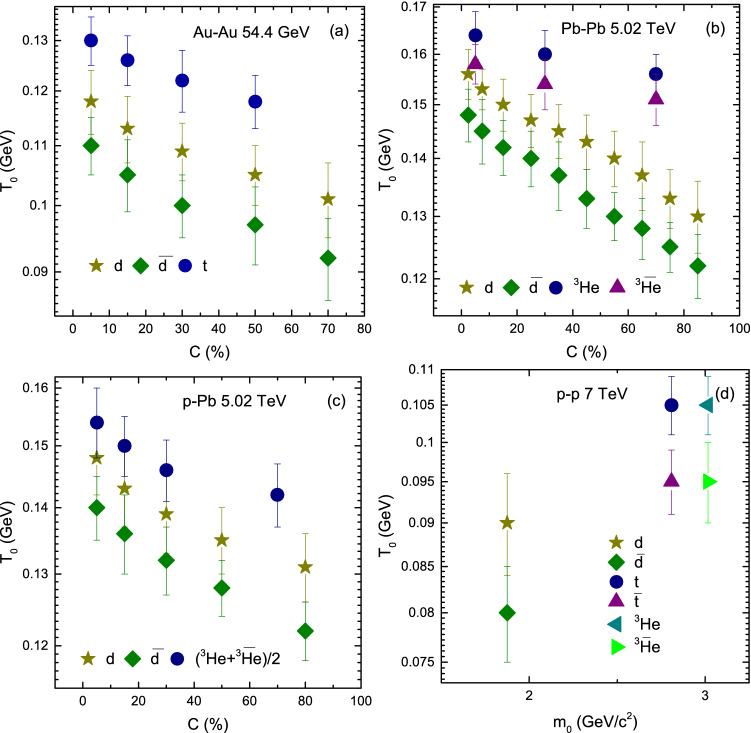
(**a–c**) Variation of $T_0$ with centrality and (**d**) variation of $T_0$ with $m_0$ for *d*, ${\bar{d}}$, *t*, $\bar{t}$, $^3{\text{He}}$ and $\bar{^{3}{\text{He}}}$ or $(^3{\text{He}}+\bar{^3\text{He}})/2$ in Au–Au, Pb–Pb, p–Pb and p–p collisions.

Figure 5 shows the variation trend of parameters with centrality (mass). Panels a–d show the results for Au–Au collisions at 54.4 GeV, Pb–Pb collisions at 5.02 TeV, p–Pb collisions at 5.02 TeV and p–p collisions at 7 TeV respectively. Panels a–c show the dependence of $T_0$ on centrality, and panel d shows the dependence of $T_0$ on $m_0$. The types of particles are represented by different symbols. In Fig. 5, panels a–c, one can see that *d*, ${\bar{d}}$, *t*, ${\bar{t}}$, $^3{\text{He}}$ and $\bar{^{3}{\text{He}}}$ in all collisions (Au–Au, Pb–Pb and p–Pb ) results in larger $T_0$ in central collisions which decrease towards periphery. The reason behind this is, in central collisions, large number of participants involve in interaction and the collisions are more violent that results in higher degree of excitation of the system and the kinetic freezeout temperature is high. However, the collisions become less violent as the centrality decreases and less number of participants involve in the interactions which results in comparatively low kinetic freezeout temperature. This is in agreement with Refs.^18–23^, but in disagreement with Refs.^24–27^.

Figure 5 includes Panel a *d*, ${\bar{d}}$, and *t*; Panel b *d*, ${\bar{d}}$, *t* and ${\bar{t}}$; Panel c *d*, ${\bar{d}}$ and $(^3{\text{He}}+\bar{^{3}{\text{He}}})/2$; and Panel d *d*, ${\bar{d}}$, *t*, ${\bar{t}}$, $^3{\text{He}}$ and $\bar{^{3}{\text{He}}}$. In some panels some particles and their anti-particles are missing due to the unavailability of data. In panel a one can see that $T_0$ is larger for *t* than both of *d* and ${\bar{d}}$ due to its mass. While *d* and ${\bar{d}}$ has the same mass but *d* freezeout earlier than ${\bar{d}}$. Similarly in panel b *d*, ${\bar{d}}$, $^3{\text{He}}$ and $\bar{^{3}{\text{He}}}$, the mass of $^3{\text{He}}$ and $\bar{^{3}{\text{He}}}$ is larger than *d* and ${\bar{d}}$, therefore they freezeout earlier than *d* and ${\bar{d}}$, but *d* and $^3{\text{He}}$ freezeout earlier than ${\bar{d}}$ and $\bar{^{3}{\text{He}}}$ respectively, while in panel c $(^3{\text{He}}+\bar{^{3}{\text{He}}})/2$ has larger $T_0$ than *d* and ${\bar{d}}$ whereas *d* freezeout earlier than ${\bar{d}}$, and in Panel d $^3{\text{He}}$ and $\bar{^{3}{\text{He}}}$, as well as *t* and ${\bar{t}}$ freezeout earlier than *d* and ${\bar{d}}$, and the values for $^3{\text{He}}$ and *t*, and $\bar{^{3}{\text{He}}}$ and ${\bar{t}}$ are respectively the same. Basically, the formation of light nuclei occur by the coalescence of nucleons with similar momenta. In the present work, we believe that the coalescence of nucleons is larger for *d*, *t* and $^3{\text{He}}$ compared to their anti-particles and therefore $T_0$ is larger for light nuclei than for their anti-nuclei. Furthermore, we observed that $^3{\text{He}}$, and *t*, and $\bar{^{3}{\text{He}}}$ and ${\bar{t}}$ freezeout at the same time. In our opinion this is due to the isospin symmetry at high energies which occurs in nearly identical masses (e.g. triton and helion) where an up quark is replaced by a down quark. In addition, $T_0$ in Pb–Pb is larger than in Au–Au and in the later, it is larger than in p–p collisions which shows its dependence on the cross-section of interacting system. However $T_0$ is larger in p–Pb than in Au–Au collisions because the center of mass energy for p–Pb is 5.02 TeV which is very larger than the center of mass energy of Au–Au collision (54.4 GeV), and this may reveal its dependence on energy.

**Figure 6:**
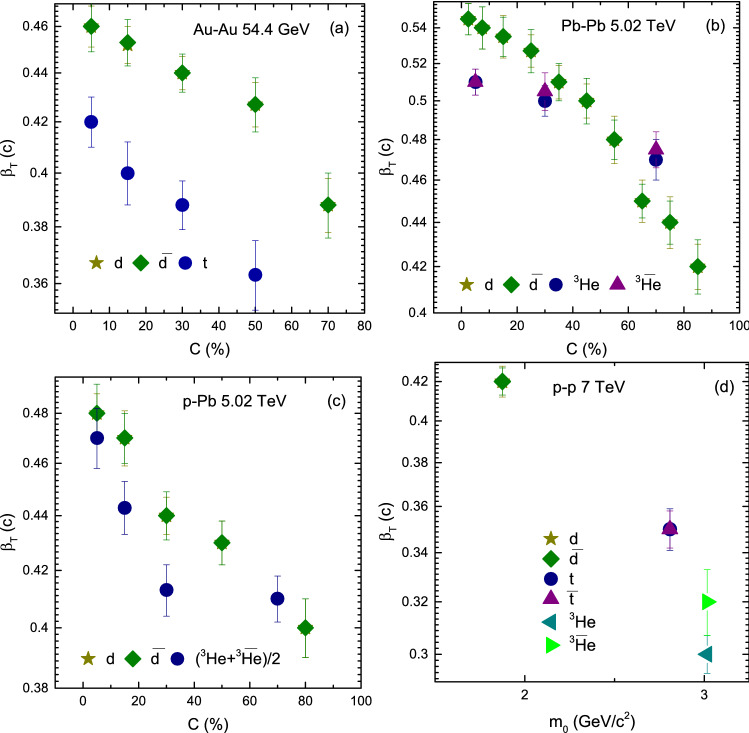
(**a–c**) Variation of $\beta _T$ with centrality and (**d**) variation of $\beta _T$ with $m_0$ *d*, ${\bar{d}}$, *t*, $\bar{t}$, $^3{\text{He}}$ and $\bar{^{3}{\text{He}}}$ or $(^3{\text{He}}+\bar{^{3}{\text{He}}})/2$ in Au–Au, Pb–Pb, p–Pb and p–p collisions.

Figure 6 is similar to Fig. 5, but shows the dependence of $\beta _T$ on centrality in panels a–c, while panel d shows its dependence on $m_0$. It can be seen that $\beta _T$ decrease from central to peripheral collisions due to large number of participants in central collision that experience more violent squeeze and results in a rapid expansion of the system. While this expansion becomes steady from central to periphery due to decreasing the participant nucleons which results in comparatively weak squeeze. Furthermore, $\beta _T$ is mass dependent. Greater the mass of the particle is, smaller the value of $\beta _T$. $\beta _T$ for nuclei and anti-nuclei is the same. Besides, $\beta _T$ shows dependence on the cross-section of interacting system. Larger the cross-section of interacting system, larger the $\beta _T$ is. However $\beta _T$ is slightly larger in p–Pb collisions than in Au–Au collisions due to the effect of very large center of mass energy of p–Pb than Au–Au collisions.

**Figure 7:**
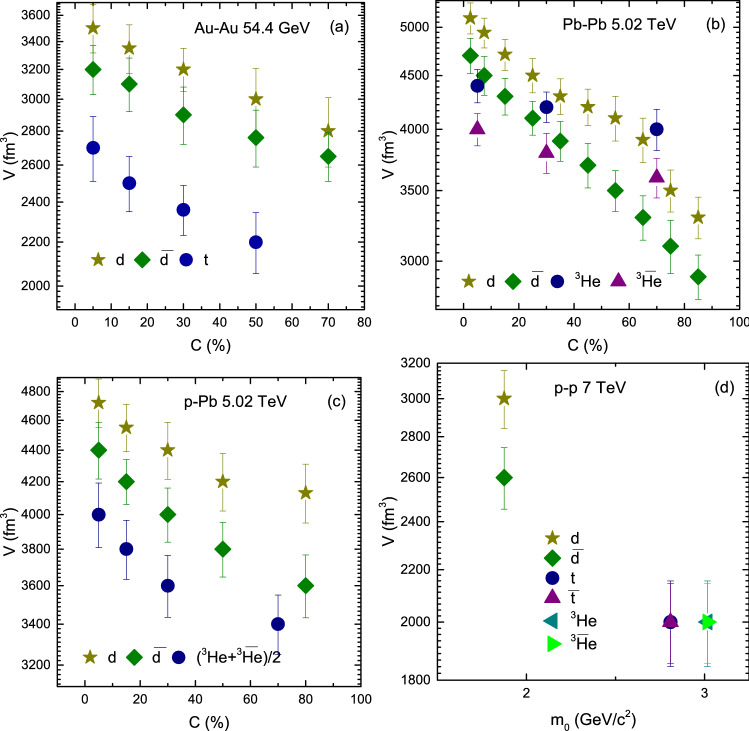
(**a–c**) Variation of $\beta _T$ with centrality and (**d**) variation of $\beta _T$ with $m_0$ for *d*, ${\bar{d}}$, *t*, ${\bar{t}}$, $^3{\text{He}}$ and $\bar{^{3}{\text{He}}}$ or $(^3{\text{He}}+\bar{^{3}{\text{He}}})/2$ in Au–Au, Pb–Pb, p–Pb and p–p collisions.

Figure 7 is similar to Fig. 6, but shows the dependence of *V* on centrality (mass). It can be seen that *V* decreases from central to peripheral collisions in panel a–c, as the number of participant nucleons decreases from central collisions to periphery depending on the interaction volume. The system with more participants reaches to equilibrium quickly due to large number of secondary collisions by the re-scattering of partons, which decreases towards periphery and the system goes away from equilibrium state. In addtion, *V* for deuteron and anti-deuteron is larger than that of triton and anti-triton as well as from helion and anti-helion. The parameter *V* for nuclei is larger than their anti-nuclei due to larger coalescence of nucleons for the nuclei than for their anti-nuclei. In case of triton and anti-triton and helion and anti-helion, V of triton and helion, and anti-triton and anti-helion are respectively the same. Besides, *V* is larger in Pb–Pb collisions that the rest, and in Au–Au collisions as well as p–Pb collisions it is larger than in p–p collisions which shows the dependence of *V* on the cross-section of interacting system. However *V* is larger in p–Pb than in Au–Au collisions. We think that this is due the effect of very higher center energy of p–Pb collisions compared to Au–Au collisions, because higher energy corresponds to longer evolution time which may lead to larger partonic system.

**Figure 8:**
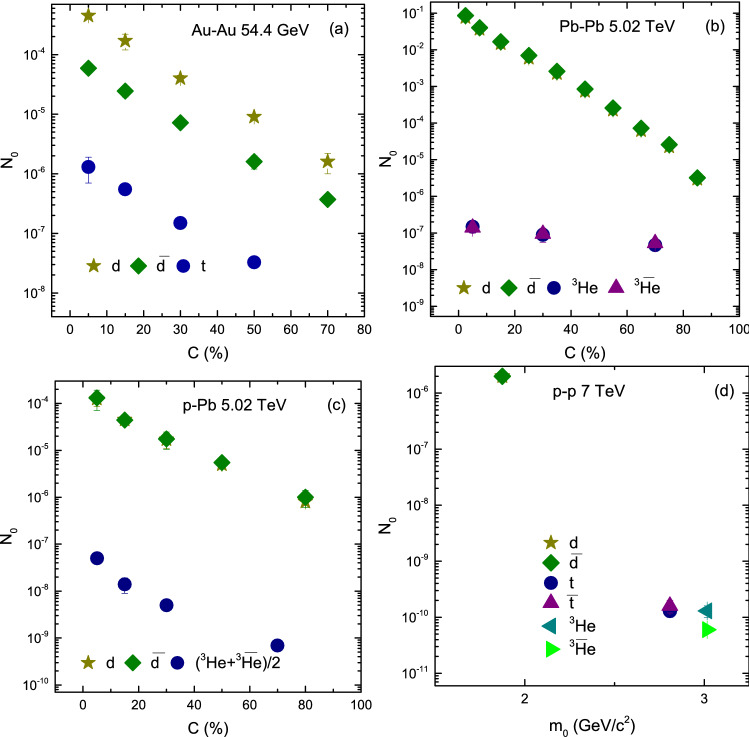
(**a–c**) Dependence of $N_0$ on centrality and (**d**) Dependence of $N_0$ on $m_0$, for *d*, ${\bar{d}}$, *t*, ${\bar{t}}$, $^3{\text{He}}$ and $\bar{^{3}{\text{He}}}$ or $(^3{\text{He}}+\bar{^{3}{\text{He}}})/2$ in Au–Au, Pb–Pb, p–Pb and p–p collisions.

Figure 8a–c show the dependence of $N_0$ on centrality. One can see that $N_0$ decrease with decreasing centrality. Furthermore, the parameter $N_0$ depends on mass of the particle. In panels a–d the parameter $N_0$ for deuteron and anti-deuteron are larger than triton, and in (a) it is larger for deuteron than anti-deuteron due to large coalescence of deuteron, while in panels b–d the parameter $N_0$ for nuclei and their anti-particles are the almost same. In general, the parameter $N_0$ is the same for nuclei and their anti-nuclei. The parameter $N_0$ for triton and helion, and anti-triton and anti-helion is the same due to the isopin symmetry. In deed $N_0$ is only a normalization factor and the data are not cross-section, but they are proportional to the volumes of sources of producing various particles. Therefore it is significant to study $N_0$ dependence.

**Figure 9:**
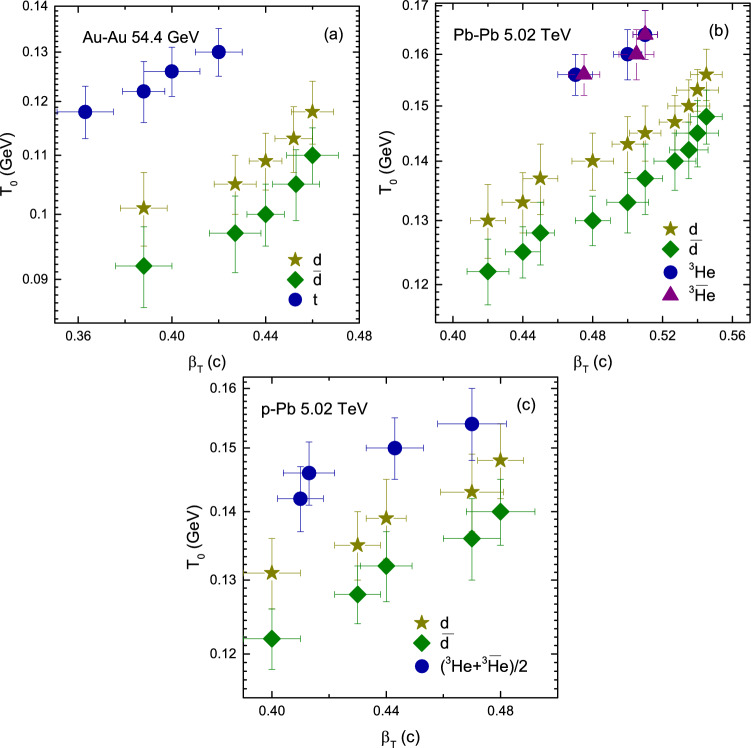
Variation of $T_0$ with $\beta _T$.

Figure 9 shows the variation of $T_0$ with $\beta _T$. It is observed that central collisions correspond to larger $T_0$ and $\beta _T$. The correlation between $T_0$ and $\beta _T$ is positive. This is in agreement with Ref.^23^ and in disagreement with Ref.^25^. In panel a the correlation of $T_0$ and $\beta _T$ is larger for triton and that of deuteron is larger than anti-deuteron. In panel b the correlation of $T_0$ and $\beta _T$ is larger for helions than deuterons and that of deuteron is larger for than anti-deuteron. Similarly in panel c the helions has larger correlation between $T_0$ and $\beta _T$ than deuteron and anti-deuteron, and for deuteron it is larger than anti-deuteron. In general, the massive particles has larger correlation between of $T_0$ and $\beta _T$, and the particles has larger correlation than their anti-particles due to less coalescence of anti-particles.

**Figure 10:**
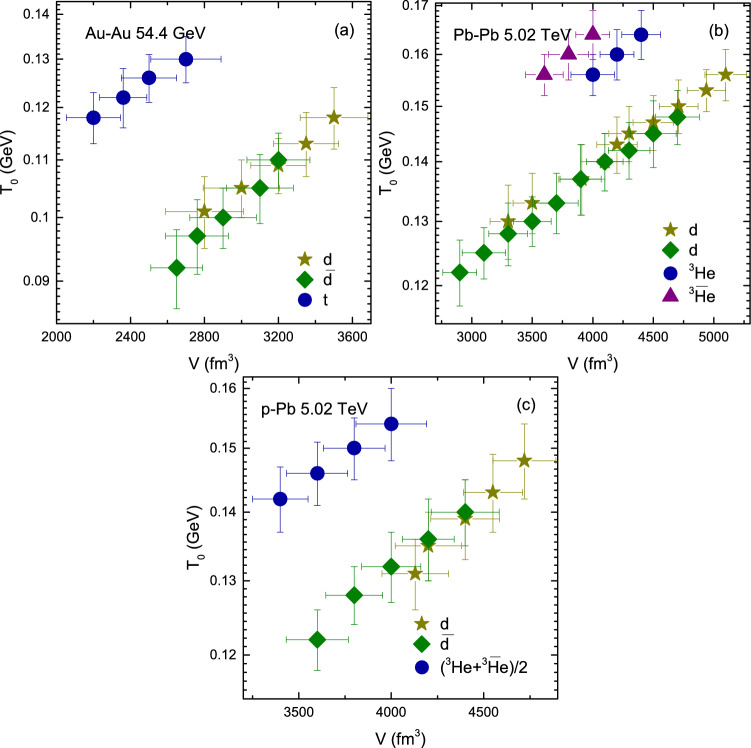
Variation of $T_0$ with *V*.

Figure 10 shows the variation of $T_0$ with *V*. It is observed that central collisions correspond to larger $T_0$ and *V*. The correlation between $T_0$ and *V* is positive. In panel a the correlation of $T_0$ and *V* is larger for triton, while the correlation of $T_0$ and *V* for deuterons is slightly larger than anti-deuteron. In panel b the correlation of $T_0$ and *V* is larger for helions than deuterons, and that of helions and deuterons is slightly larger for than their anti-particles. Similarly in panel c the helions has larger correlation between $T_0$ and *V* than deuteron and anti-deuteron and deuteron has larger correlation of $T_0$ and *V* than anti-deuteron. In short the massive particles has larger correlation between of $T_0$ and *V*, and the particles has larger correlation than their anti-particles due to less coalescence of anti-particles.

## Conclusions

The main observations and conclusions are summarized here.

- The transverse momentum spectra of light nuclei and their anti-nuclei produced in Au–Au, Pb–Pb and p–Pb and produced in inelastic p–p collisions in different centrality intervals are analyzed by the BGBW model. The model results show an agreement with the experimental data in the special $p_T$ range measured by the STAR and ALICE Collaborations.
- Kinetic freezeout temperature is larger for triton and helions and their anti-particles than deuteron and anti-deuteron due to their mass. While helions and tritons have the same value of Kinetic freezeout temperature due to isospin symmetry, and dueteron, triton and helion freezeout earlier than their anti-particles respectively due to large coalescence of nucleons for the light nuclei than anti-nuclei.
- Kinetic freezeout temperature decrease from central to peripheral collisions due to the decrease of participant nucleons in the peripheral collisions which lead to the decrease in the degree of excitation of the system.
- Transverse flow velocity increases from peripheral to central collisions due the reason that the collisions become more violent in central collisions which also expands the system rapidly.
- The kinetic freezeout volume decreases from central to peripheral collisions and the system reaches quickly to equilibrium state due to large number of secondary collisions by the re-scattering of partons in central collisions that decreases towards periphery. In addition, The normalization constant is larger in central collisions than in peripheral collisions.

## Data Availability

The data used to support the findings of this study are included within the article and are cited at relevant places within the text as references.
